# Synergistic effects of yeast and plant growth-promoting bacteria on Tobacco growth and soil-borne disease suppression: evidence from pot and field experiments

**DOI:** 10.3389/fpls.2024.1489112

**Published:** 2024-11-01

**Authors:** Kai Teng, Yu Zhou, Hui Mao, Xianjun Long, Sheng Zhang, Jingjing Ma, Delong Meng, Huaqun Yin, Yunhua Xiao

**Affiliations:** ^1^ College of Bioscience and Biotechnology, Hunan Agricultural University, Changsha, China; ^2^ Hunan Tobacco Company Xiangxi Autonomous Prefecture Corporation, Jishou, China; ^3^ School of Minerals Processing and Bioengineering, Central South University, Changsha, China; ^4^ Center for the Creation of Chinese Herbal Medicine Varieties, Yuelushan Laboratory, Changsha, China

**Keywords:** *Pichia sp*., plant growth-promoting bacteria, Tobacco growth, soil microbial community, soil properties

## Abstract

**Background:**

Tobacco (*Nicotiana tabacum* L.) is an important economic crop, and the use of plant growth-promoting bacteria (PGPB) to enhance its growth and suppress soil-borne diseases has garnered considerable research interest. However, the potential of yeast to augment the growth-promoting and disease-suppressing effects of PGPB on tobacco remains unclear.

**Methods:**

This study investigated the effects of *Pichia* sp. microbial fertilizer (J1), PGPB-*Klebsiella oxytoca* microbial fertilizer (ZS4), and their composite fertilizer (JZ) on tobacco growth indexes, soil properties, and soil microbial community through a pot experiment. Additionally, field experiments were conducted to further assess the efficacy of the composite microbial fertilizer on tobacco growth and the incidences of soil-borne diseases, including tobacco bacterial wilt (TBW) and tobacco black shank (TBS).

**Results and discussions:**

In the pot experiment, application of the microbial fertilizers significantly enhanced soil organic matter (OM), total nitrogen (TN), total phosphorus (TP), total potassium (TK), available phosphorus (AP), and available potassium (AK) levels. Compared to the control group (CK), J1, ZS4, and JZ microbial fertilizers significantly promoted tobacco growth, and the composite microbial fertilizers demonstrated superior to the individual microbial fertilizers. We found that the application of microbial fertilizer led to significant alterations in the structure and composition of the bacterial and fungal communities based on the high-throughput sequencing of 16S rRNA and internal transcribed spacer (ITS) regions. The bacterial and fungal diversity indexes showed a decreasing trend. Key microorganisms such as *Sphingomonas*, *Kitasatospora*, *Nitrosospira*, *Mortierella*, and *Trichoderma* were identified as influential in regulating soil physicochemical parameters to enhance tobacco growth. Functional prediction further demonstrated a significant increase in the relative abundances of certain enzymes, including Alkaline phosphatase, 1-aminocyclopropane-1-carboxylate deaminase (ACC deaminase), and Peroxidase, as well as antimicrobial substances like Tetracycline, Isoquinoline alkaloid, and Phenylpropanoids, following inoculation with the fertilizer. Besides, field experiments revealed that the JZ fertilizer significantly promoted tobacco growth and reduced the incidence of TBW and TBS, indicating its potential for further application in tobacco cultivation.

## 1 Introduction

Tobacco (*Nicotiana tabacum* L.) is an essential economic crop in China, and its yield and quality are affected by various factors, including the ecological environment, climatic conditions, and soil fertility (Tang et al., 2020). In recent years, with the expansion of tobacco cultivation, the excessive application of chemical fertilizers and pesticides has led to soil acidification, hardening, and nutrient imbalance and has damaged the soil microbiological system, which further affects tobacco growth and aggravates the pest and disease problems, resulting in decreased tobacco yields and quality (Yu et al., 2020; Chen et al., 2023; Ramadhan et al., 2024). Soil-borne diseases caused by bacteria or fungi occur frequently in crops, and vascular diseases such as tobacco bacterial wilt (TBW), black shank (TBS), and root rot (TRR) are prevalent in tobacco. Tobacco bacterial wilt and black shank are the two major soil-borne diseases caused by *Ralstonia solanacearum* and *Phytophthora nicotianae*, respectively (Ma et al., 2018). These diseases cause severe economic losses, resulting in tobacco nutrient depletion, root necrosis, tobacco leaf wilt, and reduced growth vigor (Wei et al., 2024). Therefore, it is essential to explore and apply alternative technologies to reduce the use of chemical fertilizers and alleviate these problems effectively.

Microbial fertilizers are a class of inoculants containing active microorganisms that are safe, efficient, and environmentally friendly (Kour et al., 2020). As a kind of green fertilizer, microbial fertilizer relies on the life activities of microorganisms to provide crops with directly absorbable nutrients, thus promoting plant growth and increasing yields. Microbial fertilizers are enriched with a large number of active microorganisms, and their application significantly increases the number and activity of beneficial microorganisms in the soil, especially plant-growth-promoting bacteria (PGPR). PGPR are a class of plant growth-promoting soil bacteria that promote plant growth by colonizing the roots of plants (Gouda et al., 2018). Through their metabolic activities, these bacteria improve soil physicochemical properties, decompose organic matter, and increase the content of nutrients such as nitrogen (N), phosphorus (P), and potassium (K) (Fasusi et al., 2021). In addition, PGPR secrete enzymes, phytohormones, antibiotics, and antimicrobial compounds during their metabolism, which not only improve the soil environment and promote plant growth but also induce the expression of resistance genes in plants and inhibit the proliferation and spread of pathogens (van Loon, 2007; Elshahat et al., 2016). For example, β-glucosidase plays an essential role in the activation of phytohormones, secondary metabolism, and carbon cycling (Ahmed et al., 2017). Antibiotics such as Penicillin and Cephalosporin are known to possess antipathogenic activity (Pichichero and Zagursky, 2014; Shahbaz, 2017). As plant antitoxins, terpenoids exhibit antifungal activity against *Cladosporium cucumerinum* and *Phytophthora infestans* (Chen et al., 2019).

PGPB has attracted much attention as a potential microbial inoculant in contemporary agriculture. Common strains in PGPB include *Azotobacter*, *Bacillus*, *Pseudomonas*, *Brevibacillus*, and *Klebsiella*, which promote tobacco growth and control various plant diseases through different mechanisms (Subhashini et al., 2016; Han, 2022). For example, *Bacillus* can induce plant systemic resistance, significantly reduce the incidence of TBS, and increase the fresh weight and plant height of tobacco (Han et al., 2016). *Pseudomonas* and *Brevibacillus* have the ability to reduce disease and produce antibiotics when applied as seed treatments (Ali et al., 2022). *Klebsiella oxytoca* exhibits a variety of plant growth-promoting properties, including the production of indole-3-acetic acid (IAA), 1-aminocyclopropane-1-carboxylate deaminase (ACC deaminase), which can significantly promote aboveground and root growth of mung bean seedlings (Buddhi et al., 2014). In addition, the strain can induce resistance in tobacco to the soft rot pathogen (Guerrieri et al., 2020). Yeasts can synthesize growth factors such as amino acids, vitamins, and enzymes to provide the nutritional support required for plant growth (Mukherjee et al., 2020). Its antimicrobial properties mainly release antimicrobial compounds such as mycobacteria and iron carriers, effectively reducing tobacco root and stem rots (Petkova et al., 2022). Some reports have revealed that yeast composite microbial fertilizers promote plant growth more than individual microbial fertilizers. For example, a composite microbial fertilizer of *Pichia farinose* FL7 with *Pseudomonas farinosa* and the composite microbial fertilizer of *Saccharomyces cerevisiae* with *Bradyrhizobium japonicum* significantly promoted the growth of soybean (Zhu, 2012; Zveushe et al., 2023). However, the potential of yeast to augment the growth-promoting and disease-suppressing effects of PGPB on tobacco remains unclear.

*K. oxytoca* ZS4 was isolated from rhizospheric soil of *Boehmeria nivea* L. in Huayuan County, Hunan province (China), with plant growth-promoting traits (Liu et al., 2021). *Pichia* sp. J1 was isolated from waste leachate at a waste transfer station in Changsha City, Hunan province (China). This strain exhibits synergistic interactions with lactic acid bacteria and other microorganisms, effectively enhancing their growth and metabolic activity (Zhang et al., 2021b). This study investigated the effect of the addition of J1, ZS4, and mixed (JZ) microbial fertilizer on the growth of tobacco (*Nicotiana tabacum* L.) through a pot experiment. Through further field experiments, we examined the tobacco applied with JZ microbial fertilizer and compared it with the control group (without microbial fertilizer), emphasizing the analysis of the growth indexes as well as the disease indexes of TBW and TBS. The research focused on (1) investigating the effect of microbial fertilizers on tobacco growth, (2) assessing the specific effects of microbial fertilizer application on tobacco rhizosphere soil microbial communities and soil physicochemical factors, and (3) exploring the relationship between critical microbial species and tobacco growth and disease resistance. This study could provide a scientific basis for dealing with the problem of declining tobacco yield, as well as new solutions for improving the efficiency of tobacco growing.

## 2 Materials and Methods

### 2.1 Experimental materials

The potting soil was obtained from the 0-15cm layer of the farmland around Hunan Agricultural University in Changsha, China (113.80°E, 28.18°N). The soil was naturally air-dried, sieved to remove plant residues and stones, and then passed through a 2 mm sieve for the potting test. The initial physicochemical properties of the soil were as follows: the pH was 5.61, and the contents of organic matter (OM), total nitrogen (TN), total phosphorus (TP), total potassium (TK), available phosphorus (AP), and available potassium (AK) were 5.740 g/kg, 0.820 g/kg, 0.130 g/kg, 14.780 g/kg, 1.570 mg/kg, and 96.340 mg/kg, respectively. The microbial fertilizers tested were yeast (*Pichia* sp.) microbial fertilizer J1, PGPB (*Klebsiella oxytoca*) microbial fertilizer ZS4, and *Pichia* sp. and *Klebsiella oxytoca* composite microbial fertilizer JZ. Fermentation Conditions: J1 microbial fertilizer: The carbon source is bran, and the nitrogen source is soybean meal, with a mass ratio 6:4. The solid-to-liquid ratio is 1:1.75. The inoculation of *Pichia* sp. J1 is 5%, with fermentation conducted at 30°C for nine days. The resulting effective viable cell count is 3.6 × 10^9^ CFU/g. ZS4 microbial fertilizer: The carbon source is bran, and the nitrogen source is soybean meal, with a mass ratio of 4:6. The solid-to-liquid ratio is 1:1.25. The inoculation of *Klebsiella oxytoca* ZS4 is 3%, with fermentation performed at 35°C for seven days. The resulting effective viable cell count is 1.1 × 10^11^ CFU/g. JZ composite microbial fertilizer: This blend of J1 and ZS4 microbial fertilizer in a fresh weight ratio of 1:1. These strains have not been used in commercial formulations, just for research purposes. They were provided by the Microbial Conservation Laboratory, School of Mineral Processing and Bioengineering (Central South University, China). J1, ZS4, and JZ microbial fertilizers were not commercially available and were used only for this study. The variety of tobacco (*Nicotiana tabacum* L.) used in the pot experiment was “Yunyan 203”, provided by Changsha Tobacco Bureau Company of Hunan Province (China).

### 2.2 Pot experiment

Four treatment groups were established for the pot experiment: control group without microbial fertilizer (CK group); applied J1 microbial fertilizer (J1 group); applied ZS4 microbial fertilizer (ZS4 group); and applied JZ composite microbial fertilizer (JZ group). Six replicates were set up for each treatment group, resulting in a total of 24 pots for the four treatment groups. When *N. tabacum* seedlings reached 3 - 5 true leaves, seedlings of similar size and health condition were selected and transplanted in pots (20.5 cm × 23.5 cm) containing 1.8 kg of soil each, one plant per pot. We mixed microbial fertilizer and soil thoroughly at 1:100 and applied it evenly around the roots of the tobacco plants. The control group applied a mixture of bran and soybean meal without microbial inoculation, and no other chemical fertilizers were added in the experiment to ensure consistency in each experimental condition. Subsequently, the tobacco plants were placed outdoors for natural growth and were watered with 300 mL every three days. we determined the growth indexes of tobacco. Soil samples (5.0 g) were collected near the roots (2-4 cm), and visible plant residues and stones in the soil were removed, then air-dried and crushed to a particle size of less than 0.147 mm (Xiao et al., 2018). One part of the soil samples was used to determine the physicochemical properties of the soil, and the other was stored in a -80°C freezer for microbiological studies.

### 2.3 Field experiment

The field experiment site was located in Daoyi Science and Technology Park, Huayuan County, Xiangxi Tujia and Miao Autonomous Prefecture, Hunan Province (25°15′38.8″N, 109°15′32.5″E, Altitude: 530 m). The climate of the region is subtropical monsoon, with an average temperature of 13.9°C, an annual sunshine length of 1600-1800 h, and an annual rainfall of 1400-1800 mm. Two treatment groups were set up for the experiment: the control group without microbial fertilizer (CK) and the group with mixed microbial fertilizer (JZ). Three blocks were set up for each treatment group, and the blocks were designed in a randomized arrangement. The area of each block was about 150 m^2^, and about 200 tobacco plants were planted in each block. The planting density of tobacco was 125 cm between rows and 60 cm between plants, and protected rows were set around. At the time of transplanting, the tobacco seedlings in the JZ group were treated with microbial fertilizer by hole application, while the CK group was treated with a mixture of bran and soybean meal without microbial inoculation; at the time of the doughnut stage, the JZ group was supplemented with microbial solution, while the CK group was supplemented with fresh water. The number of colonies of microbial fertilizer applied to each tobacco seedling was controlled at 10×10^9^ CFU/g. During the tobacco growing period, both groups regularly applied chemical fertilizers, with irrigation methods consisting of rainfed irrigation and supplementary irrigation. Measurement of tobacco growth indexes at maturity and disease surveys for TBW and TBS.

### 2.4 Measurement of soil physicochemical properties

Soil and ddH_2_O were mixed at a ratio of 1:1.25, and soil pH was measured using a pH meter. (PHS-3E, Shanghai Yidian Scientific Instrument Co., Ltd., China). The soil OM content was determined by the potassium dichromate oxidation method, the soil TN content was determined by the Kjeller method, and the soil AK was extracted with ammonium acetate and determined by a flame photometer (Li et al., 2017). The soil TP content was determined by molybdenum-antimony colorimetry after H_2_SO_4_-HClO_4_ digestion (Ding et al., 2024). The soil AP content was determined by the ammonium fluoride-hydrochloric acid extraction method (Zhang et al., 2021a). The soil TK content was determined by the NaOH alkali melting method and flame photometer (Xu et al., 2018).

### 2.5 Measurement of growth indexes and disease indexes of tobacco

After the tobacco was harvested, the number of leaves per plant was recorded; the largest leaf of each plant was selected, leaf length and width were measured with a tape measure, and the maximum leaf area of the tobacco was calculated; the distance from the bottom of the stem to the top growing point of the plant was measured with a tape measure as plant height, and the circumference of the base of the stem was measured as stem circumference; stems and leaves were weighed on an electronic balance as aboveground fresh weight (Shang et al., 2023). The tobacco bacterial wilt and black shank disease index of each plant were determined based on the Chinese national standard GB/T 23222-2008, as described by (Gao et al., 2015).

### 2.6 DNA extraction and Illumina high-throughput sequencing

The extraction and high-throughput sequencing of rhizosphere soil microbial DNA were performed by Beijing Baimark Biotechnology Co. The V3-V4 region of the 16S rRNA gene was amplified with primers 338F (5’-ACTCCTACGGGGAGGCAGCA-3’) and 806R (5’-GGACTACHVGGGGTWTCTAAT-3’). The ITS1 region of the 18S RNA gene was amplified with primers ITS1 (5’-CTTGGTCATTTAGAGGAAGTAA-3’) and ITS2 (5’-GCTGCGTTTTAGGAAGTAA-3’). PCR reaction conditions were as follows: initial denaturation at 95°C for 3 min, denaturation at 95°C for 30 s, annealing at 55°C for 30 s, extension at 72°C for 45 s for 27 cycles, and a final extension at 72°C for 10 min. The sequencing raw data has been submitted to the SRA database on the NCBI website, and the accession numbers are PRJNA1153728 and PRJNA1153733.

Raw Reads were filtered using Trimmomatic v0.33 to detect and remove sequences with quality scores below 20. Then, the sequences were denoised and chimerically filtered using UCHIME v4.2 to obtain valid reads, which were further clustered into Operational Taxonomic Units (OTUs) using an identity threshold of 97%. Finally, the OTUs are assigned a classification using the RDP classifier to ensure a confidence level of at least 50%.

### 2.7 Molecular ecological network construction and characterization

In this study, we utilized the relative abundance of bacterial and fungal OTUs in soil to construct a phylogenetic molecular ecological network (PMEN) through a random matrix theory (RMT)–based approach (Zhang et al., 2023a). This network construction method automatically identifies key OTUs and determines the network’s topological properties. The low-frequency OTUs in the samples are filtered out, and the high-frequency OTUs are retained. In our study, five or more OTUs in six sample replicates were retained for network construction. Finally, the visualization of network graphs and calculating network topology parameters were implemented using Gephi 0.10.1. Network parameters such as network density, average path length, average clustering coefficient, and modularity index were shown. Molecular ecological networks consist of many nodes (OTUs) and edges, and multiple nodes can form a module. The roles of nodes in a module are characterized by Within-module connectivity (Zi) and Participation Coefficient (Pi) (Tao et al., 2018). Based on Zi and Pi, nodes in a network can be categorized into four roles: peripheral (Zi ≤ 2.5 and Pi ≤ 0.62); connector (Zi ≤ 2.5 and Pi > 0.62); module hub (Zi > 2.5 and Pi ≤ 0.62); and network hub (Zi > 2.5 and Pi > 0.62). Nodes that play the roles of connectors, module hubs, and network hubs can serve as key taxa for maintaining network structure and functionality. We utilized ZiPi diagrams to find key species in microbial association networks. ZiPi diagrams were completed in Origin2022 and the Wekemo Bioincloud (https://www.bioincloud.tech).

### 2.8 Functional predictions

PICRUSt2 combines existing open-source tools to predict the genome of 16S rRNA gene sequences of environmental samples. Predicted functions of the metagenome in the samples based on several gene family databases, including Kyoto Encyclopedia of Genes and Genomes (KEGG) homologs (KOs) and Enzyme Commission Numbers (EC numbers) using PICRUSt2. The PICRUSt2 methodology consisted of phylogenetic localization, hidden state prediction, and sample gene and pathway abundance tables. The OTU sequences and abundances were used as inputs, and gene/enzyme and KEGG pathway abundances were used as predicted outputs. The accuracy of metagenome predictions could be verified by comparing the results of differential abundance tests of 16s rRNA-predicted metagenome with MGS data (Douglas et al., 2020).

### 2.9 Statistical Analysis

A completely randomized experimental design was used with six replicates per treatment. All data except microbiological data were statistically analyzed using IBM SPSS Statistics 26. Where Analysis of variance (ANOVA) and comparison of means were performed using Tukey’s multiple range test at *p* < 0.05. Venn plots were utilized to demonstrate the relationship between the number of OTUs in the different treatment groups. Differences in the structural composition of microbial communities were shown using a Non-metric multidimensional scaling (NMDS) analysis based on the Bray-Curtis distance. The species diversity and evenness in the microbial community were assessed using Shannon and Pielou indexes, and the Chao1 and ACE indexes were calculated to assess the species richness in the community. Analysis of species composition in microbial communities using species abundance histograms and chord diagrams. LDA Effect Size (LEfSe) analysis at the genus level was performed using LDA threshold > 3, *p* < 0.05 as a screening criterion to identify significantly different microorganisms between groups. Random Forest Analysis was utilized to assess the extent of the contribution of key microbes in rhizosphere soils. The relationship between microorganisms and soil physicochemical properties was described using redundancy analysis (RDA). Correlation analysis was performed to show Spearman’s correlation coefficients between soil physicochemical properties, tobacco growth indexes, and microbial communities to characterize the relationship between these variables. Analysis of Similarities (ANOSIM) was utilized to characterize the structural differences of the predicted functional genes. The above mapping was completed in Origin 2022 and the Wekemo Bioincloud (https://www.bioincloud.tech), utilizing Adobe Illustrator 2022 for graphic editing.

## 3 Results

### 3.1 Soil physicochemical properties

The physicochemical properties of the rhizosphere soil of tobacco were shown in **Table 1**. The content of OM (5.89-7.29 g/kg), TN (0.75-0.95 g/kg), and TP (0.09-0.31 g/kg) differed significantly (*p* < 0.05) among the different treatment groups, and significantly (*p* < 0.05) higher in JZ and ZS4 than in CK. The soil content of OM, TN, and TP in the JZ group significantly (*p* < 0.05) increased by 23.77%, 26.67%, and 244.44%, respectively, compared to the CK group. There was no significant (*p* < 0.05) difference in AP (1.55-2.29 mg/kg), AK (95.40-106.86 mg/kg), and TK (15.14-16.52 g/kg) contents among the four treatment groups, but J1, ZS4, and JZ were higher than CK. Also, soil pH was significantly (*p* < 0.05) lower in all three groups than CK.

**Table 1:** Soil physicochemical properties and growth indexes of four groups in the pot experiment.

	Parameter	CK	J1	ZS4	JZ
Soil physicochemical properties	pH	5.84 ± 0.14a	5.33 ± 0.14c	5.19 ± 0.09c	5.58 ± 0.18b
	OM(g/kg)	5.89 ± 0.77b	6.31 ± 0.61b	6.63 ± 0.34a	7.29 ± 0.47a
	AP (mg/kg)	1.55 ± 0.83a	1.82 ± 0.46a	1.93 ± 0.38a	2.29 ± 0.75a
	AK (mg/kg)	95.40 ± 12.81a	106.86 ± 16.17a	101.15 ± 14.45a	101.23 ± 6.04a
	TN(g/kg)	0.75 ± 0.03b	0.90 ± 0.03a	0.93 ± 0.04a	0.95 ± 0.07a
	TP(g/kg)	0.09 ± 0.02b	0.11 ± 0.07b	0.29 ± 0.02a	0.31 ± 0.01a
	TK(g/kg)	15.14 ± 2.23a	16.52 ± 1.63a	16.38 ± 1.26a	16.44 ± 1.00a
Growth indexes	Plant height (cm)	18.05 ± 1.52c	19.05 ± 5.93c	22.10 ± 6.41bc	34.23 ± 12.15a
	Number of leaves (pc)	9.50 ± 1.87cd	10.83 ± 1.17abc	12.00 ± 1.41ab	12.33 ± 1.75a
	Aboveground fresh weight (g)	14.08 ± 1.89c	22.24 ± 6.87bc	38.28 ± 8.14a	41.86 ± 13.73a
	Maximum leaf area (cm^2^)	88.99 ± 6.79b	114.37 ± 27.02b	160.94 ± 20.96a	162.89 ± 56.14a

CK represents tobacco without microbial fertilizer, J1, ZS4, and JZ represent tobacco with J1 microbial fertilizer, ZS4 microbial fertilizer, and JZ composite microbial fertilizer, respectively. Each value in the table is the value of six replications, and the value indicates the mean ± standard deviation; different lowercase letters in each line indicate that the difference is significant at *p* < 0.05 according to Tukey’s multiple range test.

### 3.2 Growth indexes of tobacco in pot experiment

The tobacco growth indexes of the four treatment groups were shown in **Table 1**. The picture of the growth of the tobacco plant was shown in **Supplementary Figure S1**. The growth indexes of tobacco were higher in the JZ, ZS4, and J1 groups than in the CK group, with the JZ group having the highest indexes. The plant height, number of leaves, aboveground fresh weight, and maximum leaf area of tobacco in the JZ group were significantly (*p* < 0.05) increased by 89.64, 29.79, 197.73, and 83.04%, respectively, and those of tobacco in the ZS4 group by 22.44, 26.32, 171.87, and 80.85%, respectively, when compared with those of CK.

### 3.3 Overview of the rhizosphere soil microbial communities of tobacco

#### 3.3.1 Structure and diversity of rhizosphere soil microbial communities

**Figure 1:**
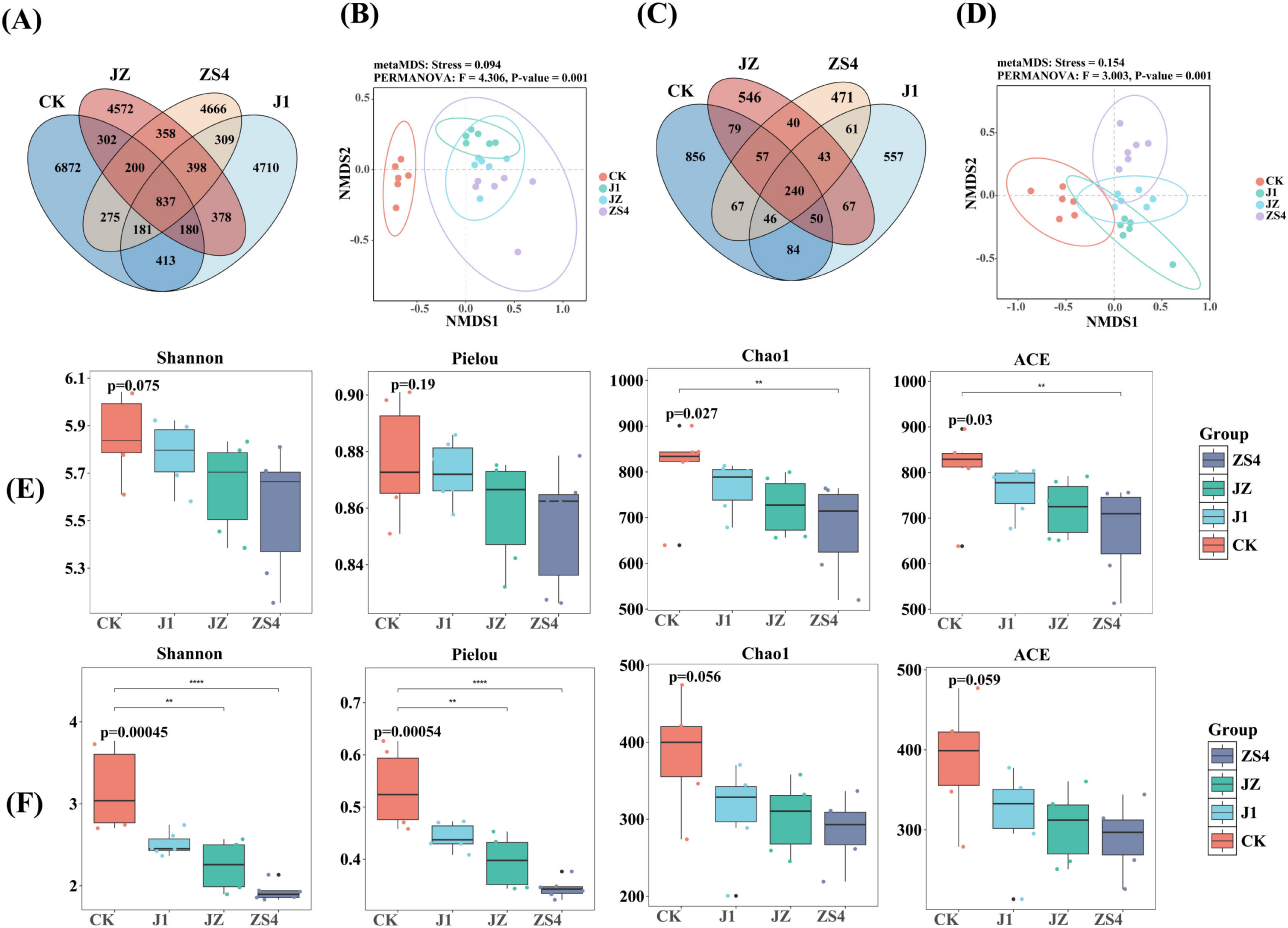
Structure and diversity of microbial communities. **(A)** Venn diagram of the bacterial community; **(B)** Non-metric multidimensional scaling (NMDS) analysis of the bacterial community; **(C)** Venn diagram of the fungal community; **(D)** Non-metric multidimensional scaling (NMDS) analysis of the fungal community; **(E)** Shannon index, Pielou index, Chao1 index and ACE index of the bacterial community; **(F)** Shannon index, Pielou index, Chao1 index and ACE index of the fungal community. Significance levels are as follows: ** , and **** indicated *p* < 0.01, and 0.0001 respectively.

As shown in the Venn diagram, there were 24651 bacterial OTUs obtained from the four groups of tobacco rhizosphere soils with 837 core OTUs, while CK, J1, ZS4, and JZ had 6872, 4710, 4666, and 4572 unique OTUs, respectively (**Figure 1A**). The fungal community had 240 core OTUs among 3264 OTUs, while CK, J1, ZS4, and JZ had 856, 557, 471 and 546 unique OTUs, respectively (**Figure 1C**). NMDS analysis showed that the sample points of different treatment groups were dispersed from each other, indicating that the similarity between these samples was relatively low and that there was a significant (*p* < 0.05) difference in microbial community structure between different treatment groups. There were significant differences between the CK and J1, ZS4, and JZ groups in the community structure of bacteria (**Figure 1B**) and between the CK and ZS4 groups in the community structure of fungi (**Figure 1D**). The α-diversity indexes of bacteria were shown in **Figure 1E**. There was no significant (*p* > 0.05) difference in Shannon, Pielou indexes among the four treatment groups, but Chao1 and ACE indexes were significantly (*p* < 0.05) higher in CK than in ZS4 group. The α-diversity indexes of fungi were presented in **Figure 1F**. There was no significant *(p* < 0.05) difference in Chao1 and ACE indexes among the four treatment groups, but the Shannon, Pielou indexes of the CK group were significantly (*p* < 0.05) higher than those of the ZS4 and JZ groups. The diversity of soil microbial communities and the number of OTUs were the greatest in the CK group.

#### 3.3.2 Composition of rhizosphere soil microbial communities

**Figure 2:**
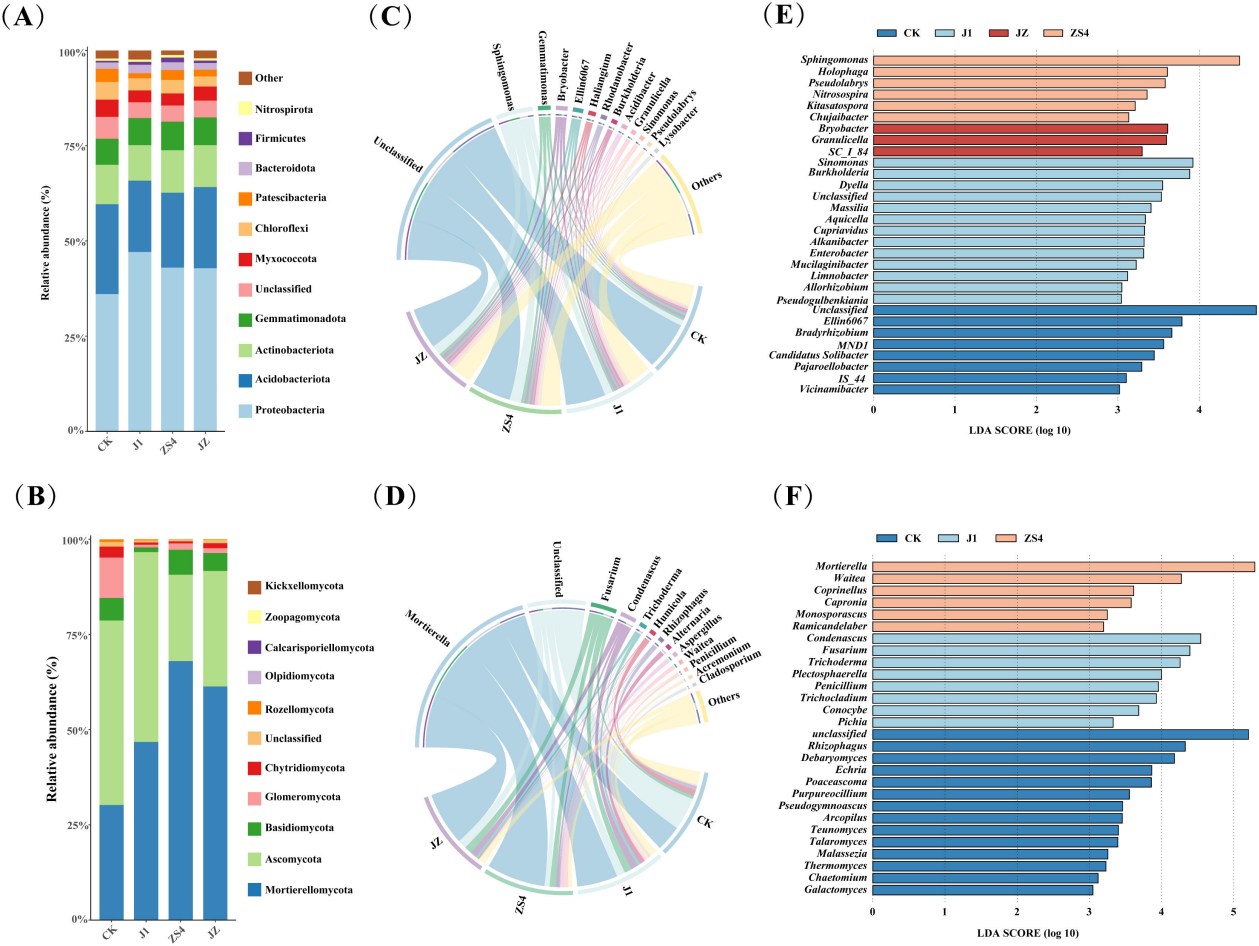
Composition of microorganisms and Linear discriminant analysis effect size (LEfSe). **(A)** The abundance of bacterial communities at the phylum level; **(B)** The abundance of fungal communities at the phylum level; **(C)** Chord diagrams show the composition of bacterial communities at the genus level; **(D)** Chord diagrams show the composition of fungal communities at the genus level; **(E)** LEfSe analysis of bacterial communities; **(F)** LEfSe analysis of fungal communities.

Soil microbial community composition was investigated and analyzed in four treatment groups, and it was found to be different in different treatment groups. The dominant bacterial phyla in the four treatment groups were Proteobacteria, Acidobacteriota, Actinobacteriota, Gemmatimonadota, and Myxococcota. The relative abundance of Proteobacteria and Gemmatimonadota was increased in J1, ZS4, and JZ groups compared to CK (**Figure 2A**). The dominant fungal phyla include Mortierellomycota, Ascomycota, Basidiomycota, Glomeromycota, and Chytridiomycota. The relative abundance of Mortierellomycota and Glomeromycota was increased in J1, ZS4, and JZ compared with CK (**Figure 2B**). As shown from the chord diagram of the bacteria (**Figure 2C**), the predominant bacterial genera common to the four treatment groups were mainly *Sphingomonas*, *Gemmatimonas*, *Bryobacter*, *Ellin6067*, and *Haliangium*. Compared with CK, the relative abundance of *Sphingomonas*, *Gemmatimonas*, and *Bryobacter* increased in the J1, ZS4, and JZ groups. Interestingly, we did not find *Klebsiella oxytoca* in all groups. Fungal community analysis revealed the dominance of *Mortierella*, *Fusarium*, and *Condenascus* in all four groups. However, the relative abundance of *Mortierella* and *Fusarium* was lower in the CK group than in the JI, ZS4, and JZ groups. The J1 group showed other prominent fungal genera, including *Alternaria* and *Trichoderma*. In contrast, the ZS4 group was characterized by the predominance of *Waitea* and *Aspergillus*. In the JZ group, other dominant fungal genera were *Trichoderma* and *Penicillium*. The dominant fungal genera in the CK group were identified as members of *Mortierella*, *Humicola*, and *Rhizophagus* (**Figure 2D**). In addition, among the other genera, we found that the relative abundance of *Pichia* in the J1 and JZ groups was 1.97% and 0.16%, respectively.

To further explore the differences in soil microbial community composition between tobacco without and with microbial fertilizer application, LEfSe analysis was conducted at the genus level, and key microorganisms that differed significantly across treatment groups were identified. The principal bacterial genera in the LEfSe analysis results were as follows: in the CK group, the genera were *Bradyrhizobium*, *Candidatus Solibacter*, and *Pajaroellobacter*; in the J1 group, there were *Sinomonas*, *Burkholderia*, *Dyella*; the ZS4 group had *Sphingomonas*, *Holophaga*, and *Pseudolabrys*, and in the JZ group were *Granulicella*, *Bryobacter*, and *SC_I_84* (**Figure 2E**). In the soil fungal community, genera differentially enriched in the CK group were mainly *Rhizophagus*, *Debaryomyces*, and *Echria*; in the J1 group were *Condenascus*, *Fusarium*, and *Trichoderma*; in the ZS4 group were *Mortierella*, *Waitea*, and *Coprinellus*, and interestingly was that there were no significantly different fungi in JZ (**Figure 2F**).

#### 3.3.3 Network interactions of rhizospheric soil microbial communities

**Figure 3:**
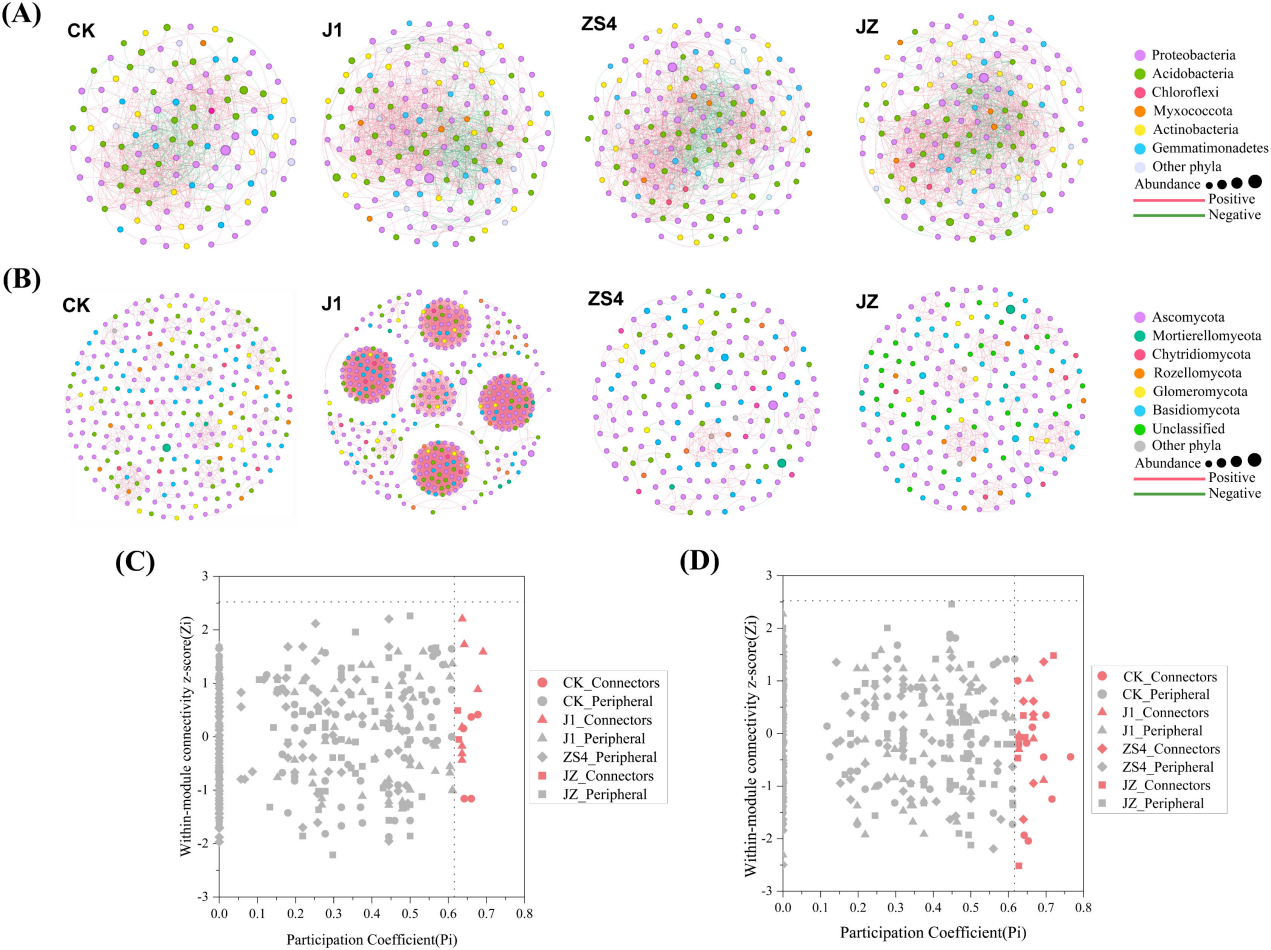
Co-occurrence network analysis of microorganisms. **(A)** Co-occurrence network plots of bacterial communities; **(B)** Co-occurrence network plots of fungal communities; **(C)** ZiPi graph of bacterial networks; **(D)** ZiPi graph of fungal networks.

We constructed microbial networks to describe symbiotic interactions at the OTU level to understand better the interrelationships between soil microbial communities of microbial fertilizer-applied and non-microbial fertilizer-applied plants. Co-occurrence network analysis at the soil bacterial community phylum level is shown in **Figure 3A**. Proteobacteria, Acidobacteria, Chloroflexi, Gemmatimonadetes, Actinobacteria, and Myxococcota accounted for a large proportion of the network in the four treatment groups and was influential in constructing the network of bacteria. The co-occurring networks at the level of soil fungal community phyla were presented in **Figure 3B**. Ascomycota, Mortierellomyeota Chytridiomycota, Basidiomycota, Glomeromycota, and Rozellomycota made up a large portion of these four networks, and these microorganisms played a critical role in constructing the networks. **Supplementary Table S1** listed the topological parameters of the co-occurrence network for bacteria and fungi. There are fewer nodes and edges in group CK (117 nodes and 728 edges) than in groups J1 (144, 1173), ZS4 (157, 1292) and JZ (157, 1311). The average clustering coefficient of 0.223 for CK was lower than that of groups J1 (0.231), ZS4 (0.228), and JZ (0.320), suggesting that the aggregation of soil bacterial communities was lowest in group CK. The topological parameters showed that the soil bacterial community network was the simplest in the CK group and the most complex in the JZ group. The J1 group had more nodes and edges (396 nodes, 6753 edges) than the CK group (278, 660), ZS4 (167, 266), and JZ (196, 432). The average clustering coefficient of 0.415 for J1 was higher than the other three groups, indicating that the highest aggregation of soil fungal communities was found in the J1 group. The topological parameters revealed that the soil fungal community network in the J1 group was the most complex. However, it was simplest in the ZS4 group. The ZiPi analysis of the bacteria revealed that the CK group had 5 connectors, the J1 group had 8 connectors, and the JZ group had 2 connectors. On the contrary, the OTUs of ZS4 belong to the peripherals, which have no connectors (**Figure 3C**). The ZiPi of the fungus showed that the CK group had 9 connectors, the J1 and ZS4 groups had 6 connectors, and the JZ group had 7 connectors (**Figure 3D**). Connectors are key microorganisms that maintain the structure and function of the network. The key soil bacterial genera revealed by ZiPi analysis included *Bryobacter*, *Lysobacter*, *Sphingomonas*, and other unclassified genera; the key fungal genera were *Nigrospora*, *Penicillium*, *Plenodomus*, *Rhizophagus*, and other unclassified genera.

#### 3.3.4 Identification of key microorganisms in the rhizosphere soil

**Figure 4:**
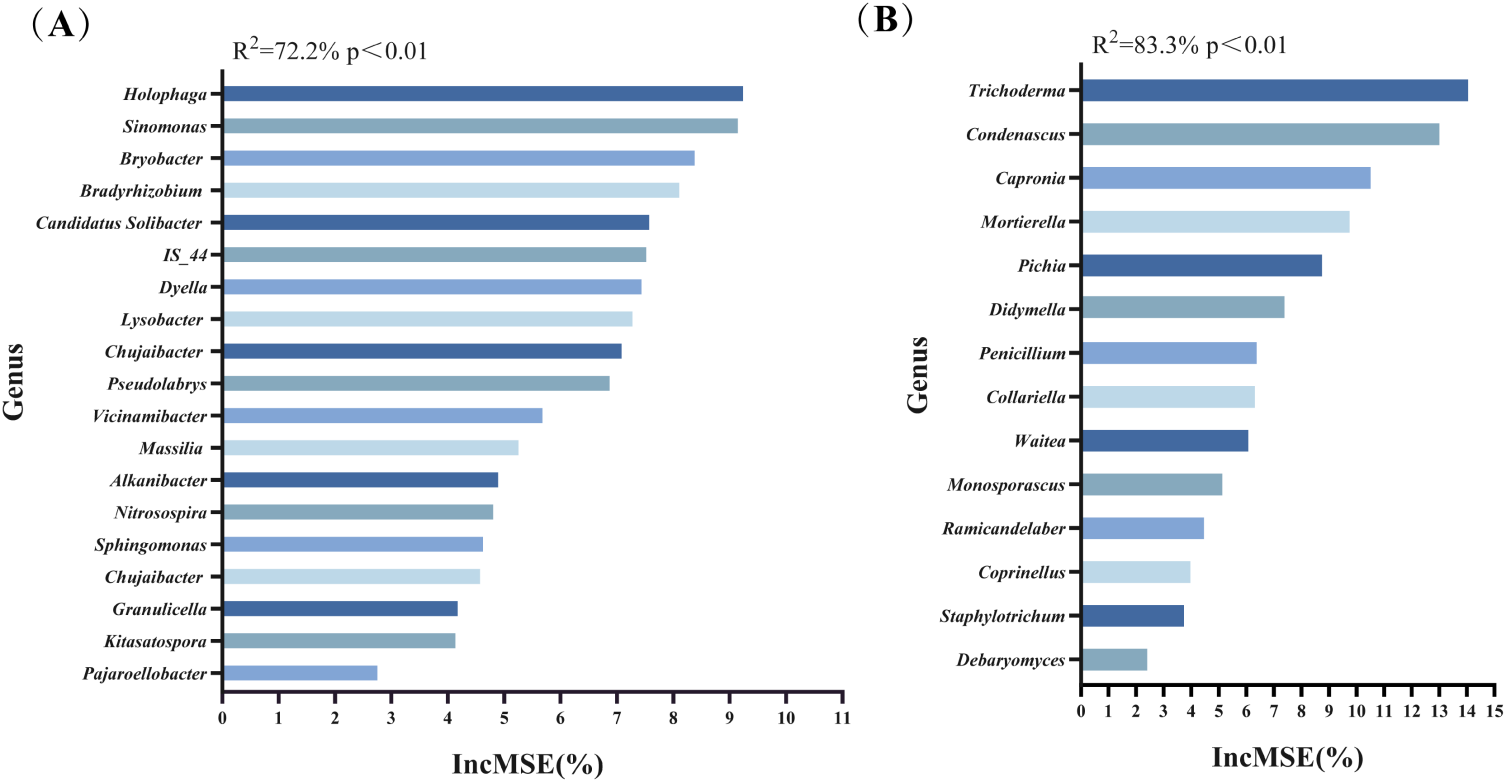
The key microorganisms at the genus level were identified by Random Forest analysis. **(A)** Key bacterial genera; **(B)** Key fungal genera.

Previously, we used LEfSe and ZiPi analyses to remove the undifferentiated microbial taxa and found the key soil microorganisms for the four treatment groups. After that, we used Random Forest analysis to rank the importance of these key genera. The larger the IncMSE, the more critical the microorganism is. These microorganisms were key genera closely associated with the soil environment and tobacco growth. **Figure 4A** presented the key genera of bacteria identified by random forest analysis, primarily *Holophaga*, *Sinomonas*, *Bryobacter*, *Bradyrhizobium*, *Candidatus Solibacter*, and others. Random forest analysis of fungi (**Figure 4B**) displayed that the key genera of fungi were mainly *Trichoderma*, *Condenascus*, *Capronia*, *Mortierella*, *Pichia*, and others.

### 3.4 Correlation analysis results

#### 3.4.1 Correlation between microbial communities and soil physicochemical factors

**Figure 5:**
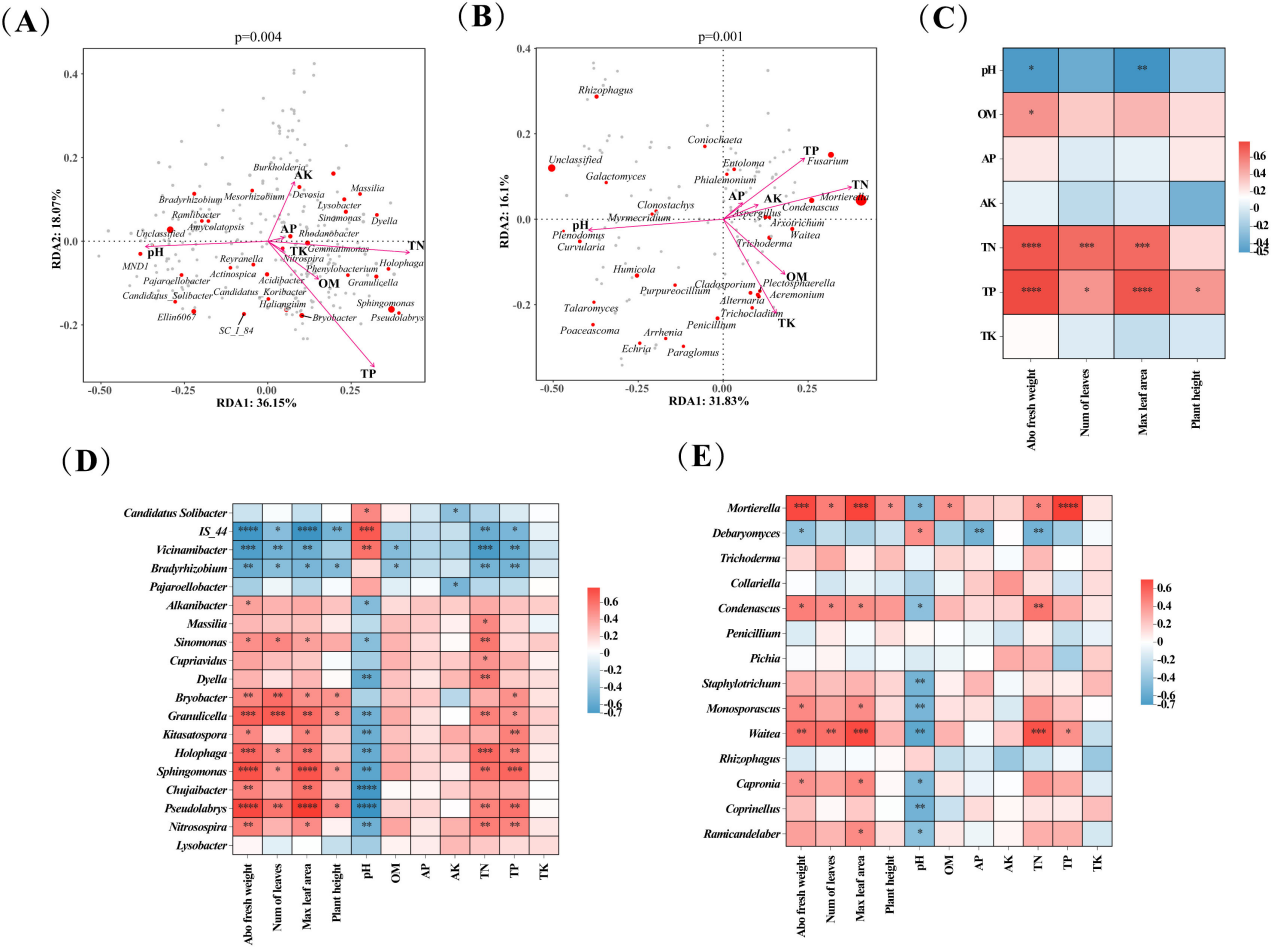
Correlation analysis of soil microbial communities, tobacco growth indexes, and soil parameters. **(A)** Redundancy analysis (RDA) plot of genus-level bacterial communities and soil parameters; **(B)** Redundancy analysis (RDA) plot of genus-level fungal communities and soil parameters; **(C)** Heat map of correlation between soil parameters and tobacco growth indexes; **(D)** Heat map of correlation of key bacteria with soil parameters and tobacco growth indexes; **(E)** Heat map of correlation of key fungi with soil parameters and tobacco growth indexes. Abo fresh weight, Aboveground fresh weight; Num of leaves, Number of leaves; Max leaf area: Maximum leaf area. Significance levels are as follows: *, **, *** and **** indicated *p* < 0.05, 0.01, 0.001, and 0.0001 respectively.

To describe the relationship between tobacco soil microbial communities and soil physicochemical factors, we performed RDA analysis at the genus level of microbial communities. The RDA plots of bacteria showed that the eigenvalues of axis 1 and axis 2 were 18.07% and 36.15%, respectively, which explained 54.22% of the relationship between the bacterial community and the soil factor (**Figure 5A**). Soil factors with high influence/correlation on bacterial communities were TP, pH, and TN. The RDA plots of fungi revealed that the eigenvalues of axis 1 and axis 2 were 16.1% and 31.83%, respectively, which explained 47.93% of the relationship between bacterial communities and soil factors (**Figure 5B**). Soil factors such as pH, TN, and TP were the essential factors influencing the soil fungal communities.

#### 3.4.2 Correlation between soil physicochemical factors and growth indexes of tobacco

The effect of soil physicochemical factors on tobacco growth was illustrated in **Figure 5C**. Soil OM content was positively associated with growth indexes of tobacco and was significantly (*p* < 0.05) positively correlated with aboveground fresh weight of tobacco. Soil TN and TP contents were positively associated with tobacco growth indexes and significantly (*p* < 0.05) correlated with aboveground fresh weight, number of leaves, and maximum leaf area of tobacco. On the contrary, soil pH, AK, AP, and TK were negatively associated with most of the growth indexes of tobacco, and pH was significantly (*p* < 0.05) negatively correlated with aboveground fresh weight and maximum leaf area of tobacco.

#### 3.4.3 Effect of key microorganisms on soil physicochemical factors and growth indexes of tobacco

The relationship between key microbial genera, soil physicochemical factors, and tobacco growth indexes was further analyzed using Spearman correlation analysis (**Figures 5D, E**), producing the following results. Most key bacteria (e.g., *Bryobacter*, *Granulicella*, *Holophaga*, *Sphingomonas*, *Pseudolabrys*, *Nitrosospira*) were significantly (*p* < 0.05) positively correlated with tobacco’s aboveground fresh weight, number of leaves, maximum leaf area, and the soil TN and TP content. However, some key bacteria (*IS_44*, *Vicinamibacter*, *Bradyrhizobium*) were significantly (*p* < 0.05) negatively correlated with these same tobacco growth indexes and soil characteristics. In addition, most key bacteria were positively correlated with the soil OM, AP, AK, and TK content but significantly (*p* < 0.05) negatively correlated with the soil pH (**Figure 5D**). Most of the key fungi were positively correlated with the growth indexes of tobacco, the soil OM, AK, AP, TN, and TP content, and they were negatively correlated with pH. For example, *Mortierella*, *Condenascus*, *Monosporascus*, and *Waitea* were significantly (*p* < 0.05) positively correlated with tobacco’s aboveground fresh weight, number of leaves, maximum leaf area, and soil TN and TP content but were significantly (*p* < 0.05) negatively correlated with soil pH (**Figure 5E**).

### 3.5 Functional prediction results

#### 3.5.1 Analysis of predicted functional genes/enzymes

Based on 16S rRNA sequences, potential functions were predicted, and the composition and differences in functional genes/enzymes of soil bacteria were explored in four treatment groups. ANOSIM analysis showed significant (*p* < 0.05) differences in predicted functional genes/enzymes in soil microorganisms between treatment groups, except for JZ/ZS4 (**Supplementary Table S2**). We identified some functional genes and enzymes associated with plant growth and disease resistance (**Table 2**). The abundance of functional enzymes (Sucrose-phosphate synthase, Chitinase, β-Glucosidase, Nitrogenase, Catalase peroxidase, Peroxidase, Alkaline phosphatase, and Nonribosomal peptide synthetase) were significantly (*p* < 0.05) higher in the J1, ZS4, and JZ groups than in the CK group., and most of the functional enzymes had greater abundance in the JZ group than in the ZS4 and J1 groups. The abundance of Tryptophan 2-monooxygenase, Urease, and ACC deaminase was significantly lower (*p* < 0.05) in the CK group than in the J1 group. The abundance of functional genes (Nitrogen fixation proteins, ATP-binding cassette transporter proteins, and MATE transporter proteins) differed significantly (*p* < 0.05) among the four treatment groups, being higher in the J1, ZS4, and JZ groups compared to the CK group.

**Table 2:** The main functional enzymes/genes associated with plant growth and disease resistance.

	Enzymes/Genes	CK	J1	ZS4	JZ
Enzymes	Sucrose-phosphate synthase	1.58E-06b	8.93E-06a	9.46E-06a	9.93E-06a
	Chitinase	3.36E-04b	3.79E-04ab	4.03E-04a	4.05E-04a
	1-aminocyclopropane-1-carboxylate deaminase	6.85E-09b	1.66E-08a	6.97E-09b	2.18E-08a
	β-Glucosidase	7.45E-03b	8.20E-03a	8.19E-03a	8.20E-03a
	Nitrogenase	1.67E-07b	5.89E-07a	5.96E-07a	4.49E-07a
	Catalase peroxidase	5.56E-04b	5.98E-04a	6.20E-04a	6.09E-04a
	Peroxidase	4.48E-04b	5.13E-04a	5.23E-04a	5.15E-04a
	Tryptophan 2-monooxygenase	7.72E-06b	2.07E-05a	4.81E-06b	6.41E-06b
	Urease	8.99E-04ab	9.32E-04a	8.01E-04b	8.04E-04b
	Alkaline phosphatase	8.75E-04b	9.62E-04a	9.61E-04a	9.75E-04a
	Nonribosomal peptide synthetase	6.66E-07b	8.16E-07ab	1.15E-06a	9.49E-07a
Genes for proteins	Nitrogen fixation proteins NifQ	1.46E-05b	2.34E-05a	1.47E-05b	1.72E-05ab
	ATP-binding cassette transporter proteins	6.71E-05b	1.02E-04a	9.88E-05b	9.46E-05b
	MATE transporter proteins	8.88E-07bc	7.18E-07c	1.47E-06a	1.18E-06b

CK represents tobacco without microbial fertilizer, J1, ZS4, JZ, represent tobacco with J1 microbial fertilizer, ZS4 microbial fertilizer, and JZ composite microbial fertilizer, respectively. Each value is based on six replicates, and different lowercase letters in each line indicate that the difference is significant at *p* < 0.05 according to Tukey’s multiple range test.

#### 3.5.2 Analysis of predicted metabolic pathways

By analyzing the composition of KEGG metabolic pathways, differences and changes in metabolic pathways of soil microbial communities in the four treatment groups could be observed (**Table 3**). We further explored changes in the abundance of genes associated with the antibiotic biosynthesis pathway (metabolism of terpenoids and polyketides and biosynthesis of other secondary metabolites). Found Penicillin and cephalosporin biosynthesis, Staurosporine biosynthesis, Isoquinoline alkaloid biosynthesis, Phenylpropanoid biosynthesis, Flavone and flavonol biosynthesis, Nonribosomal peptide structures, Brassinosteroid biosynthesis, Biosynthesis of type II polyketide backbone, Biosynthesis of terpenoids and steroids and Tetracycline biosynthesis, the abundance of 10 pathways washigher in the J1, ZS4, and JZ treatment groups than in the CK group. These pathways produce metabolites that facilitate plant growth or inhibit the growth of pathogenic microbes.

**Table 3:** Key metabolic pathways related to plant growth and disease resistance.

Metabolism	Pathway	CK	J1	ZS4	JZ
Biosynthesis of other secondary metabolites	Penicillin and cephalosporin biosynthesis	2.51E-01ab	2.38E-03b	2.56E-03a	2.49E-03ab
	Staurosporine biosynthesis	1.62E-04b	2.40E-04a	2.58E-04a	2.46E-04a
	Tropane, piperidine and pyridine alkaloid biosynthesis	3.26E-03ab	3.30E-03a	3.17E-03b	3.11E-03b
	Isoquinoline alkaloid biosynthesis	8.57E-04b	9.00E-04a	8.84E-04a	8.78E-04ab
	Phenylpropanoid biosynthesis	2.34E-03c	2.45E-03b	2.55E-03a	2.55E-03a
	Flavone and flavonol biosynthesis	2.82E-04b	3.02E-04ab	3.19E-04ab	3.34E-04a
Metabolism of terpenoids and polyketides	Nonribosomal peptide structures	1.67E-04b	2.23E-04a	2.20E-04a	2.21E-04a
	Brassinosteroid biosynthesis	1.50E-08c	3.04E-08ab	2.98E-08b	5.50E-08a
	Biosynthesis of type II polyketide backbone	1.32E-04b	1.88E-04ab	2.02E-04a	2.13E-04a
	Biosynthesis of terpenoids and steroids	2.34E-02c	2.40E-02c	2.49E-02ab	2.54E-02a
	Tetracycline biosynthesis	6.00E-03b	9.00E-03a	9.00E-03a	1.10E-02a

CK represents tobacco without microbial fertilizer, J1, ZS4, JZ, represent tobacco with J1 microbial fertilizer, ZS4 microbial fertilizer, and JZ composite microbial fertilizer, respectively. Each value is based on six replicates, and different lowercase letters in each line indicate that the difference is significant at *p* < 0.05 according to Tukey’s multiple range test.

### 3.6 Growth and disease indexes of tobacco in field experiment

A field experiment was conducted to validate further the effect of no microbial fertilizer application and application of mixed microbial fertilizer groups on tobacco growth. The photographs of tobacco plant growth in the field experiment were shown in **Supplementary Figure S2**. The growth and disease indexes of tobacco in the two treatment groups of the field experiment were presented in **Table 4**. The plant height (113.27 cm), stem girth (11.02 cm), number of leaves (14.67 pc), maximum leaf length (82.93 cm), maximum leaf width (30.25 cm), maximum leaf area (1766.58 cm^2^) of tobacco were significantly (*p* < 0.05) increased in JZ group as compared to CK. The incidence rate (13.33%) and disease index (1.48) of tobacco bacterial wilt and incidence rate (3.33%) and disease index (0.37) of tobacco black shank in the JZ group were significantly (*p* < 0.05) lower than the CK group. The relative efficacy of tobacco bacterial wilt and black shank in the JZ group was 85.71% and 66.67%, respectively, greater than that in the CK group.

**Table 4:** Growth and disease indexes of tobacco in two treatment groups of the field experiment.

	Parameter	CK	JZ
Growth indexes	Plant height (cm)	103.40 ± 6.67b	113.27 ± 7.68a
	Stem girth (cm)	9.78 ± 0.80b	11.02 ± 1.00a
	Number of leaves (pc)	12.57 ± 1.59b	14.67 ± 1.88a
	Maximum leaf length (cm)	72.10 ± 6.73b	82.93 ± 7.61a
	Maximum leaf width (cm)	23.30 ± 3.15b	30.25 ± 3.71a
	Maximum leaf area (cm^2^)	1184.66 ± 239.93b	1766.58 ± 327.76a
Tobacco bacterial wilt parameter	Incidence rate (%)	66.7a	13.33b
	Disease index	10.37a	1.48b
	Relative efficacy (%)	–	85.71
Tobacco black shank parameter	Incidence rate (%)	10.00a	3.33b
	Disease index	1.11a	0.37b
	Relative efficacy (%)	–	66.67

CK represents tobacco without microbial fertilizer and JZ represents tobacco with JZ composite microbial fertilizer. Each value is based on six replicates, growth index is expressed as mean ± standard deviation, and all other values are expressed as mean. Different lowercase letters in each line indicate significant differences at *p* < 0.05 according to Duncan’s multiple range test.

### 4 Discussion

Microbial fertilizers have been shown to promote the growth of tobacco by increasing soil fertility and reducing the incidence of soil-borne diseases, and composite microbial fertilizers have shown superior effects compared to individual microbial fertilizers, which is in accordance with the results of the present study (Ruan et al., 2020). The results of the pot experiment showed that the tobacco growth indexes of J1, ZS4, and JZ groups with microbial fertilizers were higher than that of the CK group without microbial fertilizers, and the effect of composite microbial fertilizers JZ was significantly greater than that of individual microbial fertilizers J1, suggesting that *Pichia* sp. J1 had the potential to augment the growth-promoting effects of PGPB on tobacco. In addition, further field experiments showed that tobacco in the JZ group with compound fertilizer was significantly (*p* < 0.05) higher than that in the J1 group with individual microbial fertilizer in growth promotion effect and disease resistance. These results may be related to microbial fertilizer-induced changes in soil physicochemical properties, microbial community structure, and promotion of microbial metabolic activities (Zhai et al., 2020). Therefore, it is necessary to study soil properties and soil rhizosphere microbial communities in soil-microbe-tobacco systems.

### 4.1 Microbial fertilizer application to improve soil nutrients for tobacco growth

The present study showed that OM, AP, AK, TN, TP, and TK contents were increased in J1, ZS4, and JZ groups added with microbial fertilizers compared to the CK group without microbial fertilizers (**Table 1**), which could be because the microbial fertilizers increased the nutrient content of the soil, thus promoting the growth of tobacco (Kour et al., 2020). *Pichia* sp. has an ammonia-oxidizing capacity that converts ammonia in the soil and increases plant uptakeable N (Zhang et al., 2021b). *Klebsiella oxytoca* has a phosphate solubilizing capacity and nitrogen fixation that increases the soil N and P content (Khalifa and Aldayel, 2022). Notably, we did not detect *Klebsiella oxytoca* in the later stages of the experiment. This may be because, as the microbial community evolved, *Klebsiella oxytoca* gradually disappeared, and even though it died out in the later stages, its early effects were still present (Xu et al., 2019). In addition, the contents of OM, AP, TN, and TP in the JZ group were higher than those in the J1 and ZS4 groups, which indicated that the enhancement of nutrients by composite microbial fertilizers was better than that of individual microbial fertilizers, and this also explained that the promotion effect of composite microbial fertilizers was higher than that of individual microbial fertilizers on tobacco. Correlation analysis showed that the content of OM, TN, and TP in the soil was positively correlated with aboveground fresh weight, number of leaves, maximum leaf area, and plant height of tobacco (**Figure 5C**). This might be due to the enrichment of beneficial microorganisms in the soil after the application of microbial fertilizers, which could fix nitrogen, dissolve phosphorus and potassium, and decompose organic matter, thus promoting the uptake of plant nutrients (Wei et al., 2024). As shown in **Table 1**, pH was significantly (*p* < 0.05) lower in the J1, ZS4, and JZ groups compared to the CK group. Soil pH was significantly (*p* < 0.05) negatively correlated with tobacco aboveground fresh weight and plant height (**Figure 5C**). This phenomenon could be caused by soil microorganisms such as *Klebsiella oxytoc*a, which carry out metabolic activities to secrete acids such as organic acids (Park et al., 2013; Zúñiga-Silgado et al., 2020). A decrease in soil pH can increase the mobility of essential heavy metals such as iron and magnesium in the soil, which promotes the effective uptake of these heavy metals by plants and further promotes plant growth (Zeng et al., 2011).

### 4.2 Effect of microbial fertilizers on microbial community structure and diversity in tobacco rhizosphere soil

Our results showed that applying microbial fertilizers led to changes in the structure and diversity of soil microbial communities in the tobacco rhizosphere. From **Figure 1**, soil microbial community diversity and richness decreased in J1, ZS4, and JZ groups applied with microbial fertilizers compared to CK. This may be due to the effect of *Pichia* sp. J1 and *Klebsiella oxytoca* in the soil, which makes microbial fertilizers alter the physicochemical properties of the soil and affect the microbial environment, resulting in the inability of certain microorganisms to adapt to the new conditions and reducing microbial diversity (Zhang et al., 2017; Wang et al., 2018). This study showed that the relative abundance of Proteobacteria, *Sphingomonas*, and *Mortierella* PGBRs was higher in J1, ZS4, and JZ groups than in the CK group (**Figure 2**). PGBR competed for resources with pre-existing microorganisms in the soil, which led to reduced numbers of inter-root pathogens and other harmful microorganisms. They decreased microbial diversity, promoting plant growth (Podile and Kishore, 2007). Competitive, synergistic, or mutually beneficial relationships may occur between different microorganisms, enhancing or diminishing microbial communities’ network complexity (Selim and Zayed, 2017). As revealed by the co-occurrence network analysis of microorganisms (**Figure 3**), the structural complexity of the soil microbial community network was increased in the J1, ZS4, and JZ group compared to CK, suggesting that microbial fertilizers may enhance microbial synergistic effects, which in turn promotes the growth of tobacco (Wu et al., 2024).

### 4.3 Key microorganisms promote tobacco growth through different mechanisms

Through Spearman correlation analysis, we revealed the relationship between key microorganisms, soil factors, and tobacco growth indicators. Key bacteria such as *Sphingomonas* and *Granulicella* significantly (*p* < 0.05) enhanced aboveground fresh weight, leaf number, maximum leaf area, and plant height of tobacco. They were significantly (*p* < 0.05) positively correlated with TN and TP content in soil (**Figure 5D**). Previous studies have shown that *Sphingomonas* can fix nitrogen, ammoniate, and solubilize phosphorus, which helps to increase the N and P content of the soil, which in turn promotes plant growth (Wang et al., 2024). It also regulates phytohormones such as abscisic acid, jasmonic acid, and salicylic acid, which are essential for plant development and disease defense (Mazoyon et al., 2023). *Granulicella* promotes plant growth by synthesizing IAA and extracellular polysaccharides and inhibits pathogen growth by competitively adsorbing iron from the soil, reducing the source of iron available to pathogens (Kielak et al., 2016). Our findings were consistent with these reports. In addition, *Kitasatospora* and *Nitrosospira* were significantly (*p* < 0.05) positively correlated with aboveground fresh weight and maximum leaf area of tobacco, and *Nitrosospira* was significantly (*p* < 0.05) positively correlated with soil TN (**Figure 5D**). This correlation could be attributed to *Nitrosospira*’s role as a nitrifying bacterium involved in nitrification, which promoted plant growth by modifying soil N morphology and increasing plant AN (Ayiti et al., 2022). *Kitasatospora* acts as a PGPB that produces high concentrations of IAA (Arunachalam Palaniyandi et al., 2013) or promotes plant growth by inhibiting plant pathogens through the production of antimicrobials such as propioxatins, terpentecin, and setamicin (Arunachalam Palaniyandi et al., 2013). Our study also identified some key fungi affecting tobacco growth, such as *Mortierella*, *Trichoderma*, *Condenascus*, and *Waitea*, that positively correlated with growth indicators of tobacco (**Figure 5E**). It reported that *Mortierella* and *Trichoderma* belong to plant growth-promoting fungi. They could produce phytohormone and ACC deaminase to promote the growth of tobacco, which could protect the plant from pathogens and reduce the incidence of TBW (Deng et al., 2018; Li et al., 2020). In addition, *Mortierella* improved P content in soil, which was consistent with the results of the present study, showing a significant (*p* < 0.05) positive correlation between *Mortierella* and TP (Ozimek and Hanaka, 2021).

### 4.4 Effects of predicted functional enzymes/genes and secondary metabolites on growth promotion and disease suppression in tobacco

This study investigated the role of rhizosphere microbial metabolic activities in promoting plant growth. As seen from **Table 2**, the abundance of Sucrose-phosphate synthase (SPS), ACC Deaminase, Nitrogenase, and Alkaline Phosphatase was significantly (*p* < 0.05) higher in J1, ZS4 and JZ groups with added microbial fertilizers than in the CK group (without microbial fertilizers). We speculated that these enzymes facilitate tobacco growth by participating in the cycling of carbon, nitrogen, and phosphorus in the soil and regulating the uptake of tobacco nutrients. Specifically, ACC deaminase promotes plant growth by cleaving ACC, providing essential carbon and nitrogen sources for plants and microorganisms, and reducing ethylene levels in plants (Arshad et al., 2007). Nitrogenase, encoded by the Nif gene, can fix atmospheric nitrogen (N_2_) to provide plants with a needed nitrogen source (Zhang et al., 2023b). Alkaline phosphatase catalyzes the hydrolysis of organophosphorus compounds, releasing inorganic phosphate, which can be correlated to the key microbes *Sphingomonas* and *Mortierella* for phosphate solubilization. In addition, this study found that adding microbial fertilizer significantly enhanced the activities of antioxidant enzymes in plants. The abundance of Catalase peroxidase and Peroxidase was significantly (*p* < 0.05) higher in the J1, ZS4, and JZ groups than in the CK group. These antioxidant enzymes could effectively scavenge reactive oxygen species (ROS) and reduce oxidation-induced plant cell damage, promoting tobacco growth (Khan et al., 2023). The abundance of β-glucosidase, Chitinase, and Nonribosomal peptide synthetase was higher in the treatment group with microbial fertilizer addition than in the CK group. Chitinase can degrade chitin, a significant component of the cell wall of phytopathogenic fungi, thereby defending against fungal diseases while improving plant growth and yield (Kumar et al., 2018). Nonribosomal peptide synthetase is responsible for the synthesis of nonribosomal peptides, which are peptide natural products with properties such as toxins, iron carriers, and antibiotics (Martínez-Núñez and López, 2016). MATE transporter proteins and ATP-binding cassette transporter proteins (ABC transporter proteins) had the highest abundance in the ZS4 and J1 groups, respectively. We concluded that these enzymes function in inhibiting tobacco diseases and promoting tobacco growth. MATE transporter proteins are involved in the transport of organic acids, phytohormones, and secondary metabolites and regulate plant disease resistance as well as metal detoxification; ABC transporter proteins have been shown to participate in membrane transport of endogenous secondary metabolites in plants and play an essential role in antifungal diterpene secretion and heavy metal detoxification (Liu et al., 2023). Overall, adding microbial fertilizers increased the levels of some enzymes and genes related to plant growth promotion and resistance to pathogens, thereby promoting nutrient uptake, disease resistance, and growth of tobacco.

Analysis of the predicted metabolic pathways revealed that the abundance of biosynthetic pathways of secondary metabolites of some microorganisms was higher in the treatment group where microbial fertilizers were applied than in the CK group (**Table 3**). Tetracycline biosynthesis was significantly (*p* < 0.05) higher in the J1, ZS4, and JZ groups than in the CK group. Tetracycline has been shown to have antipathogenic activity (Daghrir and Drogui, 2013). Moreover, Tetracycline plays a role in inhibiting TBS and TBW (Tang et al., 2023). Alkaloids such as Tropane, Piperidine, Pyridine, and Isoquinoline Alkaloids and flavonoids such as Flavone and flavonol exhibit a wide range of biological activities, including antiviral, antiparasitic, antimicrobial, and antioxidant properties (Sireesha et al., 2019; Zandavar et al., 2023). Phenylpropanoid and Brassinosteroid biosynthesis abundance was remarkably (*p* < 0.05) higher in the J1, ZS4, and JZ groups than in the CK group. The biosynthesis of Phenylpropanoid has been shown to inhibit *Cercospora nicotianae* and *Phytophthora nicotianae*, thereby enhancing disease resistance in tobacco (Yadav et al., 2020). Brassinolide (BL) is the essential brassinosteroid, which plays a crucial role in plant growth and development, and BL-treated wild-type tobacco was significantly more resistant to tobacco mosaic virus (TMV), *Pseudomonas syringae pv*. tabaci (Pst), and *Oidium* sp (Nakashita et al., 2003). This study found that the application of J1, ZS4, and JZ microbial fertilizers enhanced the activity of the above-mentioned antimicrobial compounds in tobacco, which reduce tobacco Soil-borne diseases by inhibiting the activity of pathogenic bacteria, thus promoting their growth, which could also explain the low incidence of TBW and TBS in the field experiment.

## 5 Conclusion

The study showed that J1, ZS4, and JZ microbial fertilizers enhanced the growth of tobacco and reduced diseases such as tobacco bacterial wilt and black shank disease, and the effect of the composite microbial fertilizers was better than that of the individual microbial fertilizers. Microbial fertilizer application increased soil OM, TN, TP, TK, AP, and AK levels and affected the structure and function of soil microbial communities. Some key bacteria (e.g., *Sphingomonas*, *Kitasatospora*, *Nitrosospira*) and key fungi (e.g., *Mortierella*, *Trichoderma*) could promote tobacco growth directly or indirectly by regulating soil factors or modulating functional enzymes/genes. In addition, some beneficial microorganisms secreted antimicrobial compounds (e.g., Tetracycline, Isoquinoline alkaloid, Phenylpropanoid) through metabolic activities to reduce tobacco diseases. Alkaline phosphatase, ACC deaminase, Peroxidase, and other enzymes, as well as Nitrogen fixation proteins NifQ and MATE transporter proteins, played essential roles in the growth of tobacco. These findings provided substantial support for enhancing tobacco cultivation efficiency, economic benefits, and environmental sustainability.

## Data Availability

The datasets presented in this study can be found in online repositories. The names of the repository/repositories and accession number(s) can be found in the article/**Supplementary Material**.
