# Supplementary material for: Synergistic effects of yeast and plant growth-promoting bacteria on Tobacco growth and soil-borne disease suppression: evidence from pot and field experiments
Source: Front Plant Sci. 2024 Nov 1;15:1489112. doi: 10.3389/fpls.2024.1489112 (PMC11563955; doi:10.3389/fpls.2024.1489112)
Supplement: Supplementary file 1 [file DataSheet1.docx]

Table S1．Topological properties of molecular ecological networks of microbial communities in different groups.

| Topological properties | Bacteria | | | | Fungi | | | |
| --- | --- | --- | --- | --- | --- | --- | --- | --- |
|  | CK | J1 | ZS4 | JZ | CK | J1 | ZS4 | JZ |
| Nodes | 117 | 144 | 157 | 157 | 278 | 396 | 167 | 196 |
| Edges | 728 | 1173 | 1292 | 1311 | 660 | 6753 | 266 | 432 |
| Modularity | 0.369 | 0.343 | 0.347 | 0.353 | 0.947 | 0.779 | 0.928 | 0.922 |
| Network diameter | 7 | 6 | 7 | 9 | 2 | 1 | 2 | 2 |
| Network density | 0.054 | 0.057 | 0.057 | 0.058 | 0.009 | 0.043 | 0.01 | 0.011 |
| Average path length | 2.197 | 2.175 | 2.193 | 2.215 | 1.003 | 1 | 1.007 | 1.002 |
| Average clustering coefficient | 0.223 | 0.231 | 0.228 | 0.320 | 0.366 | 0.415 | 0.344 | 0.351 |
| Positive correlation | 75% | 75.11% | 73.76% | 74.6% | 98.18% | 99.96% | 94.34% | 98.38% |
| Negative correlation | 25% | 24.89% | 26.24% | 25.4% | 1.82% | 0.04% | 5.36% | 1.62% |

Note: CK represents tobacco without microbial fertilizer, J1, ZS4, and JZ represent tobacco with J1 microbial fertilizer, ZS4 microbial fertilizer, and JZ composite microbial fertilizer, respectively.

Table S2. ANOSIM analyses the structure difference in predicting enzymes/genes between treatment groups.

| Predicting parameters | Group | Distance | R | P |
| --- | --- | --- | --- | --- |
| Enzymes | J1/CK | Bray-Curtis | 0.430 | 0.004 |
|  | JZ/CK | Bray-Curtis | 0.287 | 0.004 |
|  | ZS4/CK | Bray-Curtis | 0.287 | 0.008 |
|  | J1/JZ | Bray-Curtis | 0.322 | 0.01 |
|  | ZS4/J1 | Bray-Curtis | 0.239 | 0.007 |
|  | JZ/ZS4 | Bray-Curtis | 0.011 | 0.380 |
| Genes | J1/CK | Bray-Curtis | 0.346 | 0.007 |
|  | JZ/CK | Bray-Curtis | 0.280 | 0.002 |
|  | ZS4/CK | Bray-Curtis | 0.228 | 0.006 |
|  | J1/JZ | Bray-Curtis | 0.354 | 0.005 |
|  | ZS4/J1 | Bray-Curtis | 0.244 | 0.002 |
|  | JZ/ZS4 | Bray-Curtis | 0.035 | 0.271 |

Note: CK represents tobacco without microbial fertilizer, J1, ZS4, and JZ represent tobacco with J1 microbial fertilizer, ZS4 microbial fertilizer, and JZ composite microbial fertilizer, respectively. Bray-Curtis: calculates the distance between two samples. R-value: measures the magnitude of the difference between groups, the R-value ranges from -1 to 1; P-value: to determine whether there is a difference between the different groups (*p* < 0.05, the difference between groups is significant).


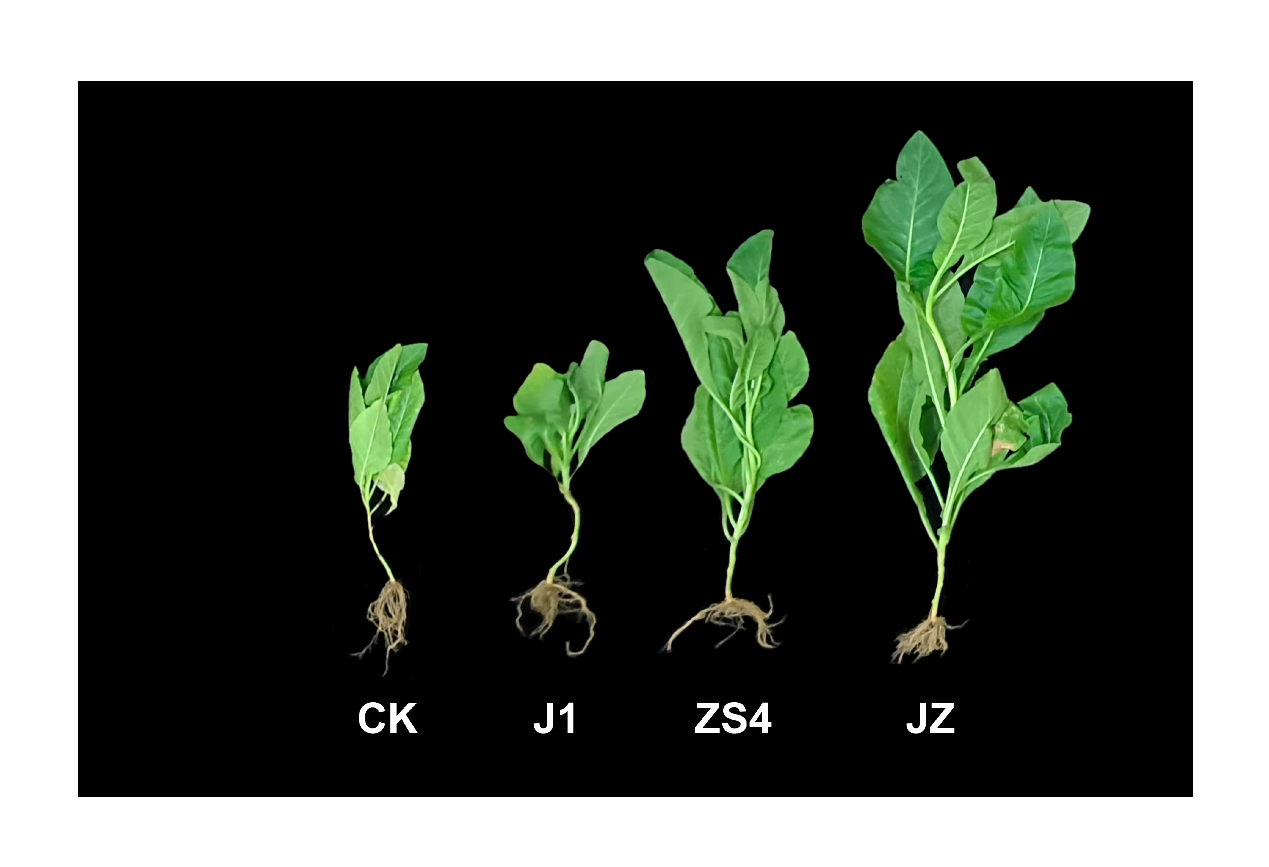


Figure S1. The growth status of four groups of tobacco plants in the pot experiment. CK represents tobacco without microbial fertilizer, J1, ZS4, and JZ represent tobacco with J1 microbial fertilizer, ZS4 microbial fertilizer, and JZ composite microbial fertilizer, respectively.


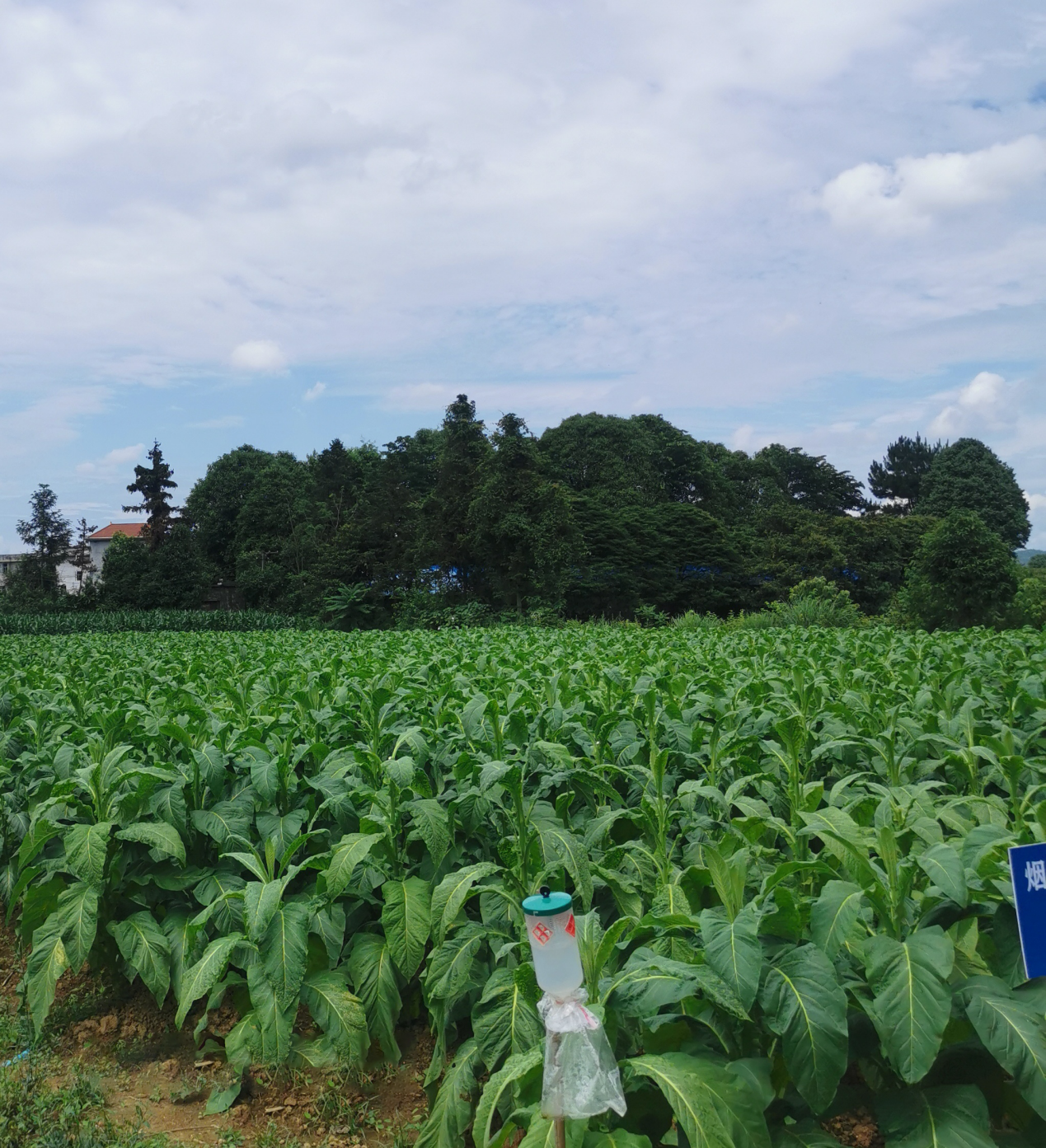


Figure S2. The growth status of tobacco plants in the field experiment.
